# Seroprevalence of Anti-Hepatitis E Virus Antibodies among Patients from a Tertiary Hospital from Northeast Romania

**DOI:** 10.3390/medicina58081020

**Published:** 2022-07-29

**Authors:** Ioana Florina Mihai, Dragos Anita, Olivia Simona Dorneanu, Catalina Mihaela Luca, Carmen Doina Manciuc, Cristian Constantin Budacu, Florin Manuel Roșu, Gheorghe Savuta, Adriana Anita, Andrei Vâţă

**Affiliations:** 1 Infectious Diseases Department, “Grigore T. Popa” University of Medicine and Pharmacy, 16 Universității Street, 700116 Iași, Romania; iordanioana06@gmail.com (I.F.M.); dmanciuc@yahoo.com (C.D.M.); manuelflorin.rosu@gmail.com (F.M.R.); andreiandrei@yahoo.com (A.V.); 2 Regional Center of Advanced Research for Emerging Diseases Zoonoses and Food Safety (ROVETEMERG), “Ion Ionescu de la Brad” University of Life Sciences, 3 Mihail Sadoveanu Alley, 700490 Iași, Romania; danita@uaiasi.ro (D.A.); epirovet@yahoo.com (G.S.); 3 Microbiology Department, “Grigore T. Popa” University of Medicine and Pharmacy, 16 Universității Street, 700116 Iași, Romania; odorneanu@yahoo.com; 4 Dento-Alveolar and Maxilo-Facial Surgery Department, “Grigore T. Popa” University of Medicine and Pharmacy, 16 Universității Street, 700116 Iași, Romania; cristibudacu@yahoo.com

**Keywords:** hepatitis E, antibodies, zoonotic transmission, endemic region

## Abstract

*Background and Objectives*. Being an enterically transmitted pathogen with a growing prevalence in developed countries, hepatitis E virus (HEV) infection remains an underdiagnosed disease in Eastern Europe. As far as Romania is concerned, only a few studies address this issue. Our goal was to estimate the prevalence of serum anti-HEV IgA/IgM/IgG antibodies in a group of patients admitted to the Clinical Hospital for Infectious Diseases “St. Parascheva” Iasi. *Materials and Methods.* The cross-sectional study consisted of enrollment of 98 patients admitted to the clinic for COVID-19 over a period of three months in 2020. *Results*. The median age in our study was 73 years, with an equal gender ratio and with a predominance of people from the urban environment (75%). The overall HEV antibody seroprevalence was 12.2%. The main risk factors associated with HEV infection were consumption of water from unsafe sources (58.3% HEV-positive patients vs. 26.7% HEV-negative patients, *p* = 0.026) and improperly cooked meat (58.3% HEV-positive patients vs. 23.2% HEV-negative patients, *p* = 0.01). Zoonotic transmission was an important criterion in our study, with patients reporting contact with pigs, poultry, rats, or other farms animals, but no significant differences were found between HEV antibody positive and negative groups. *Conclusions*. The seroprevalence rate of HEV antibodies was similar to other previous reports from our area but higher than in most European countries. The fact that HEV antibodies were detected in patients without identifiable risk factors for hepatitis E is evidence of subclinical infection as a silent threat.

## 1. Introduction

When it was discovered in 1983, the hepatitis E virus (HEV) was initially designated as “enterically transmitted non-A and non-B hepatitis” [1]; the first HEV genomic sequence was reported eight years later [2]. Hepatitis E virus is a quasi-enveloped, positive- strand RNA virus belonging to the *Hepeviridae* family. The last taxonomy structured the family into two genera: genus *Orthohepevirus* and genus *Piscihepevirus* [3]. Eight genotypes of HEV are currently described that share the same serotype. Among the five major human hepatotropic viruses (along with hepatitis A, B, C, and D viruses), the hepatitis E virus is unique, being the only one with zoonotic transmission [4]. 

Viral hepatitis E is prevalent in Asian regions, where it is considered endemic. A mathematical model estimated that hepatitis E virus leads to 20 million new infections annually in Asia and Africa, where the prevalent HEV strains are genotypes 1 and 2 [5]. A growing number of cases have also been reported from developed countries, where the prevalent HEV strains are genotypes 3 and 4, the most likely cause being zoonotic transmission [6,7]. Recently, zoonotic infections have also been evidenced by rat HEV-C [8]. Whereas the seroprevalence of HEV infection in developing countries is estimated between 30% and 80%, in developed countries, the seroprevalence is between 1% and 20% [9].

Acute liver infection caused by HEV continues to be the most neglected of all five types of viral hepatitis, with approximately 3.3 million symptomatic cases. HEV is responsible for 3.3% of all deaths due to viral hepatitis [7,10]. In Europe, hepatitis E is a self-limiting infection that lasts for several weeks and is usually asymptomatic. Symptomatic patients in developed countries are more generally men over the age of 50 with previous various liver diseases [11,12]. Jaundice may occur in about 40% of cases [13,14]. Severe forms have been observed in pregnant women or people over the age of 40, more frequently in men, who often have other coexisting diseases [15,16,17]. Immunosuppressed people are at risk of developing chronic hepatitis E infection [18]. 

Previous studies conducted in Romania revealed a seroprevalence of anti-HEV IgG Ab, ranging from 13.11% to 17.14% in northeastern counties [19,20] and between 12.5% and 13.98% in southern counties [21]. Moreover, the detection of anti-HEV IgM antibodies (9 out of 90) in patients with acute hepatitis [22] raises awareness of hepatitis E as an emerging infection in our country. 

Studies in humans are sparse; therefore, more data are needed to understand the epidemiology of HEV infection in Romania. This fact motivated the authors of this study to focus their attention on identifying persons with markers of hepatitis E infection. Thus, we performed serological tests to determine HEV (IgA/IgG/IgM) antibody seroprevalence in a cohort of patients admitted to the Infectious Diseases Hospital for other conditions. Our purpose was also to assess more detailed data, such as the possible routes of transmission, burden, or identification of particular risk factors associated with the presence of anti-HEV antibodies, to increase the awareness of HEV infection in the general population.

## 2. Materials and Methods

### 2.1. Study Design and Patients

We conducted a cross-sectional study at the “St. Parascheva” Infectious Disease Hospital in Iasi, Romania, in which were randomly enrolled adult (over 18 years old) patients (based on their chart numbers), admitted to the clinic for COVID-19, during three months (from October to December 2020). Upon enrollment in the study, patients received, in addition to informed consent, an epidemiological questionnaire designed to help outline the risk factors associated with HEV. The questionnaire included questions regarding demographic data (age, gender), place of residence (urban/rural), occupation and daily activities (contact with animals, standing water, history of travel abroad in the last year), eating habits (consumption of undercooked or raw meat products and seafood, consumption of water from unsafe sources), comorbidities, and past blood transfusions. Contact with animals was defined as the presence of domestic animals in patients’ households, and contact with standing water was defined as any source of stagnant water, including ponds, drain water, and reservoirs. Concerning eating habits, we were interested in the consumption of pork, game meat, fish, or other seafood, with an emphasis on insufficiently cooked dishes, regardless of the amount ingested. For unsafe water consumption, we included patients who reported water consumption from wells or various places, except bottled water. We also extracted demographic, anamnestic, and laboratory data from the patients’ medical charts. None reported a previously documented HEV infection.

### 2.2. Detection of HEV Antibodies 

After obtaining written informed consent from all patients, serum samples were collected and a commercially available ELISA kit was used for the detection of anti-HEV antibodies (EUROIMMUN, https://www.euroimmun.com (accessed on 10 March 2021), catalog number EI 2525-9601 *P*) for appropriate semi-quantitative in vitro determination of IgA, IgM, and IgG against hepatitis E virus. The kit contained microplate strip wells coated with recombinant target antigens of hepatitis E virus genotype 1 and 3 expressed in *E. coli.* Results were calculated by semi-quantitative assessment using the following formula: optical density (OD) ratio of the extinction of the control or patient sample over the extinction of the calibrator. The results were interpreted as recommended by the manufacturer: <0.8 for negative samples, ≥0.8 to <1.0 as borderline, and >1.1 for positive samples. Diagnostic sensitivity of the ELISA kit to anti-HEV (IgA, G and M) in patient sera was determined by the manufacturer and amounted to 100%, with a specificity of 97.8%. Subsequently, quantification of IgG anti-HEV antibodies in international units (UI/mL) was performed using the ELISA test kit (EUROIMMUN, https://www.euroimmun.com (accessed on 10 March 2021), catalog number EI 2525-9601 *G*). Liver enzymes—alanine aminotransferase (ALT), aspartate aminotransferase (AST)—were determined using an RX Imola analyzer.

### 2.3. Ethics 

The study was approved by the Ethics Committee of the hospital and was conducted in accordance with the ethical principles of the latest version of the 2013 Helsinki Declaration. All patients signed the informed consent before enrolling in the study cohort.

### 2.4. Statistical Analysis 

Correlation between demographic parameters, clinical data, and outcome was performed using Pearson’s test in XLSTAT version 2019 software (Addinsoft, Paris, Ile-de-France, France). Chi square coefficients were calculated. Statistical analysis was performed using Statistical Software for Excel (XLSTAT) version 2019. A *p* < 0.05 was considered significant.

## 3. Results

Ninety-eight patients agreed to participate in the study, and the samples were collected on the first day of hospitalization. Of the total samples taken, using the semi-quantitative IgA, IgM, and IgG anti-HEV ELISA test, 82 (83.7%) were negative, 4 (4.1%) had borderline results, and 12 (12.2%) tested positive. Using the quantitative IgG anti-HEV antibody test, all 12 patients who previously tested positive remained positive, and the rest of the patients were negative (including the four patients with previous borderline results). 

Negative samples represented group A (86 patients) and positive samples group B (12 patients). The overall HEV antibody seroprevalence was 12.2%. The demographic data of the patients are analyzed in Table 1. 

Concerning the age distribution, patients positive for HEV antibodies were older than patients negative for HEV antibodies: The median age of the patients in group B was 73 years and that of the patients in group A was 60 years (*p* = 0.019), with no gender predominance. For both groups, most patients originated from urban areas. 

Regarding the geographic distribution, most of the HEV seropositive patients originated from Iași County, respectively, Iași City (8 patients) and its surroundings (3 patients), and only one patient from Roman City in Neamt County. 

Based on the epidemiological questionnaire, we set out to assess the significant risk factors associated with hepatitis E virus infection (Table 2). 

A higher seroprevalence of anti-HEV antibodies was found in the group of patients who reported consuming water from unsafe sources (*n* = 7 (58.3%) vs. *n* = 23 (26.7%), *p* = 0.026) and in patients who reported consuming underprepared meat (*n* = 7 (58.3%) vs. *n* = 20 (23.2%), *p* = 0.01). Other associated HEV risk factors evaluated in patients with positive results were contact with pets (*n* = 8 (66.7%) versus (vs.) *n* = 49 (56.9%)) and consuming fish or other seafoods (*n* = 5 (41.7%) vs. *n* = 27 (31.4%)) or game meat, but no statistical significance was found between the two groups. The current profession was also an aspect of the questionnaire. Whereas patients in group A declared various jobs (priest, salesmen, teachers, doctors, pensioners), group B included 87.5% retired persons. 

Only two patients had a history of past blood transfusions, and both were identified as seronegative for HEV antibodies. Eight patients reported travels abroad within the last year, and were also HEV seronegative. Evaluation of the personal history of patients with HEV-positive serology highlighted three patients with heart disease (hypertension, heart failure), one patient with respiratory disease (chronic obstructive pulmonary disease), and one patient with cancer and liver metastases.

**Figure 1:**
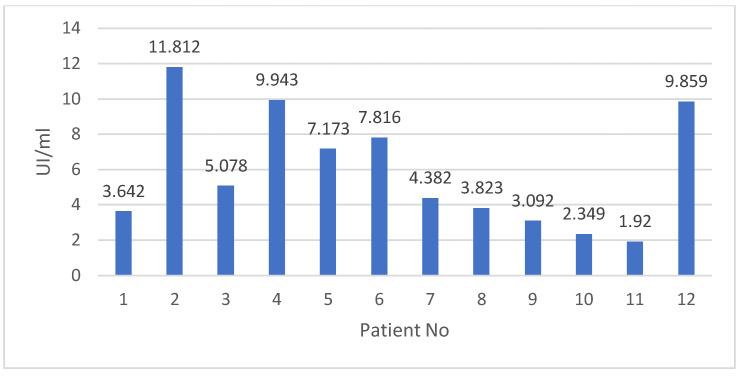
Quantitative determination of IgG anti-HEV antibodies of group B patients.

The quantitative determination of IgG anti-HEV antibodies of group B patients revealed values between 1.9 UI/mL and 11.8 UI/mL, with a median value of 4.7 UI/mL (Figure 1). Two out of three patients with values above 9 UI/mL had no identifiable risk factors for HEV infection.

The level of the transaminases was similar between the two groups (mean alanine aminotransferase (ALAT) 0.95 vs. 1.46 the upper normal limit (UNL), *p* = 0.16; mean aspartate aminotransferase (ASAT) 0.89 vs. 1.24 UNL, *p* = 0.13), but was difficult to interpret because COVID-19 (the reason for the patient’s hospitalization) is a known cause of liver damage [23].

## 4. Discussion

In recent decades, seroprevalence studies of HEV infection have shown its occurrence in non-endemic areas in people without a history of endemic travel, and HEV infection prevalence worldwide may have been previously under-reported [24,25]. The frequent and continuous detection of specific HEV in animals (domestic pigs, wild boars, and rats) [26,27,28] could make hepatitis E an emerging public health concern, particularly because there is limited information related to the risk levels, both in terms of disease incidence in the general population and possible sources of the infection [7,25]. 

Regarding the seroprevalence of anti-HEV IgG in the European countries classified as socio-economically developed, the number of HEV cases has increased in the last years. France registered an increase from 38 positive cases in 2005 to 2280 cases in 2016, the Netherlands reported in 2014 an incidence of confirmed cases five times higher than in previous years, and in Germany, HEV cases increased by 40 times in 10 years (2005–2015) [29,30]. The results of the Public Health England monitoring program of acute HEV cases in England and Wales revealed a year-on-year increase in the number of infections from 2008 (*n* = 183) through 2016 (*n* = 1243) [31]. In our study, anti-HEV (IgA, G, and M) seropositivity was estimated at 12.8%. This is a higher percentage compared to some European countries (Germany 4.8%, Belgium 7.4%, the Netherlands 3.7%, Spain 4.3%) [7], but lower compared to other European countries (16.3% in France). 

European guidelines recommend anti-HEV serology as the first-line investigation of any patient with an unexplained ALT elevation [32,33]. In our study, the ALAT levels were slightly increased and probably influenced by SARS-CoV-2 infection and anti-inflammatory drugs used in its therapy. None of the patients had ALAT > 300 IU/L or other signs of acute hepatitis.

In Romania, the first research conducted on hepatitis E infection [34] was aimed the detection of HEV infection in domesticated pigs. Further investigations confirm the potential role of the animal reservoir in the occurrence of this zoonosis, while also suggesting that the hepatitis E virus is widespread in pig farms in northeastern Romania [20] and also in the wild boar population [35]. Zoonotic transmission was an important criterion in our study; patients reported contact with pigs, poultry, rats, or other farm animals, but no significant differences were found between HEV antibody positive and negative groups.

Considering that our study included only patients hospitalized in the Clinical Hospital of Infectious Diseases Iasi and the sample size was limited, the results obtained represent only a part of the potential carriers of HEV from the NE area of Romania. We believe that our findings should be the basis for more detailed studies on the incidence of HEV infection in Romania. 

The median age of the positive patients in our study was 73 years, with no gender predominance, and with most patients originating from an urban environment (75%). The risk factors associated with HEV infection were the consumption of water from unsafe sources (58.3%, *p* = 0.026) and improperly prepared meat (58.3%, *p* = 0.01). As a result of a large host range for HEV (pig, deer, wild boar, or seafood and fish), there is a high risk of contracting hepatitis E virus from consuming undercooked products from these animals/shellfish. In this sense, one study in the Netherlands highlighted a higher seroprevalence of HEV antibodies in meat-eaters (22.8%) compared with vegetarians (13.8%) [36]. Patients over the age of 60 seem to be most at risk of presenting symptomatic infection. Identification of predisposing factors to an acute infection could target the people likely to benefit from dietary recommendations to reduce the risk of HEV infection.

A recent study published in April 2019 regarding the identification of risk factors associated with HEV seropositivity in Romania identified 46 patients (out of a total of 175 patients tested—26.3%) with positive anti-HEV IgG antibodies, with the mean age being 54.5 years old. Associated risk factors were previous blood transfusions (22.7%), immunosuppressive disorders (40.9%), and hepatitis B virus co-infection (31.1%) [37]. In our study there were no subjects who had received blood transfusion, and none of the patients were previously diagnosed with hepatitis B infection.

The present study has some limitations. The size of the studied group was relatively small, and the geographic concentration of the patients in Iasi Country makes the results less significant for the wider Northeastern Romania region. It was difficult to objectively assess the eating habits and contact with animals, and we had to rely on self-reported data. In addition, the SARS-CoV-2 infection of the patients at the time of blood sampling interfered with the interpretation of the transaminases.

Awareness of the increased incidence of HEV infection and its geographical spread should be improved. The augmented prevalence of HEV infection in non-endemic areas may change the perception of epidemiological features, and an increase in interest in this area is needed. 

## 5. Conclusions

The seroprevalence rate of HEV antibodies was similar to other previous reports from Eastern and Southern Romania, but higher than most European countries. The fact that HEV antibodies were detected in patients without identifiable risk factors for hepatitis E is evidence of subclinical infection as a silent threat. Further studies should focus on clinical and epidemiological aspects to broaden the spectrum of hepatitis E virus detection and proper diagnosis.

## Figures and Tables

**Table 1:** Demographic characteristics of the patients.

Characteristics	Group A (*n* = 86)	Group B (*n* = 12)	*p*-Value
Age—median, IQR	60 (62–81)	73 (44–70)	0.019
Female gender—*n* (%)	45 (52.3%)	6 (50%)	0.82
Urban residence	52 (60.4%)	9 (75%)	0.35

**Table 2:** Epidemiological characteristics of the patients.

Characteristics	Group A (n = 86)	Group B (n = 12)	*p*-Value
Lives with pets	49 (56.9%)	8 (66.7%)	0.68
Contact with pigs	26 (30.2%)	3 (25%)	0.71
Contact with rats	35 (40.7%)	3 (25%)	0.29
Contact with poultry	39 (45.3%)	4 (33.3%)	0.43
Contact with other farms animals	4 (4,6%)	1 (8.3%)	0.75
Contact with standing water	18 (20.9%)	2 (16.7%)	0.73
Unsafe water source	23 (26.7%)	7 (58.3%)	0.026
Consumption of undercooked meat	20 (23.2%)	7 (58.3%)	0.01
Consumption of game meat	3 (3.4%)	2 (16.7%)	0.051
Consumption of seafood	27 (31.4%)	5 (41.7%)	0.48

## Data Availability

All data generated or analyzed during this study are included in this article. Further enquiries can be directed to the corresponding authors.
